# Supplementary material for: Food safety in Thailand 4: comparison of pesticide residues found in three commonly consumed vegetables purchased from local markets and supermarkets in Thailand
Source: PeerJ. 2016 Sep 1;4:e2432. doi: 10.7717/peerj.2432 (PMC5012412; doi:10.7717/peerj.2432)
Supplement: Data S1 [file peerj-04-2432-s001.docx]

**Supplementary Data I**

**(1) Concentration of pesticides detected in Chinese kale samples bought from Local markets**

***MRL value (ppb) is given in the parenthesis.**

| **Sample No.** | **Pesticide Concentration (ppb)** | | | | | | | | | | | | |
| --- | --- | --- | --- | --- | --- | --- | --- | --- | --- | --- | --- | --- | --- |
|  | **Carba-ryl**  **(50)*** | **Carbo-furan (20)*** | **Chloro-thalonil (4000)*** | **Chlor-pyrifos (50)*** | **λ-Cyha-lothrin (1000)*** | **Cyper-methrin (1000)*** | **Delta-methrin (500)*** | **Dia-zinon (50)*** | **Dime-thoate (20)*** | **Mala-thion (3000)*** | **Meta-laxyl (2000)*** | **Metho-myl** | **Profe-nofos (10)*** |
| 1 |  |  |  |  |  |  |  |  |  |  | 40.0 |  |  |
| 2 |  |  |  |  |  |  |  | 0.08 |  |  | 0.12 |  | 341 |
| 3 |  |  |  |  |  |  |  |  |  |  | 1.12 |  |  |
| 4 |  |  |  |  |  |  |  |  |  |  | 0.12 |  | 72.1 |
| 5 |  |  |  |  |  |  |  |  |  |  | 12.7 |  |  |
| 6 |  |  |  |  |  |  |  | 0.01 |  |  | 1.13 |  |  |
| 7 |  |  |  |  |  |  |  |  |  |  | 5.5 |  |  |
| 8 |  |  |  |  |  |  |  |  |  |  | 0.16 |  | 68.5 |
| 9 |  |  |  |  |  |  |  | 0.20 | 21.4 |  | 0.50 |  |  |
| 10 |  |  |  | 7.2 |  |  |  |  | 28.3 |  | 3.33 |  |  |
| 11 |  |  |  |  |  |  |  | 0.25 | 26.5 |  | 0.36 |  |  |
| 12 |  |  |  |  |  |  |  |  | 20.0 |  |  |  |  |
| 13 |  |  |  | 37691 |  | 809 |  |  | 33.5 | 292 | 122 |  |  |
| 14 |  |  |  | 24.9 |  |  |  | 1.12 | 33.2 |  | 0.45 |  |  |
| 15 |  | 62.5 |  |  |  |  |  | 0.25 | 32.0 |  |  |  |  |
| 16 |  |  |  |  |  |  |  | 0.30 | 4.85 |  | 0.39 |  |  |
| 17 |  |  |  |  |  |  |  | 13.6 | 13.6 |  | 0.35 |  |  |
| 18 |  |  |  |  |  |  |  | 6.09 |  |  | 0.59 |  | 1993 |
| 19 |  |  |  |  |  |  |  |  | 5.8 |  | 0.08 |  | 26.6 |
| 20 | 3.61 | 0.30 |  |  |  |  |  | 21.4 | 10.5 |  | 5.7 |  | 122 |
| 21 | 3.78 |  |  |  |  | 189 |  |  | 20.1 |  | 1.10 |  |  |
| 22 | 0.75 |  |  |  |  |  |  |  | 22.5 |  | 0.62 |  |  |
| 23 | 1.29 |  |  |  |  | 5776 |  |  | 52.6 |  | 0.43 |  |  |
| 24 | 1.64 |  |  |  |  |  |  |  | 21.3 |  | 1.00 |  |  |
| 25 | 1.41 |  |  |  |  |  |  |  | 41.2 |  | 1.00 |  |  |
| 26 | 1.64 |  |  |  |  |  |  | 1.48 | 23.3 |  | 1.10 |  |  |
| 27 | 1.56 |  |  |  |  |  |  | 2.77 | 27.7 |  | 10.7 |  |  |
| 28 | 0.08 | 0.14 |  |  |  |  |  |  | 35.4 |  | 0.23 |  | 29.8 |
| 29 |  |  |  |  |  |  |  | 0.64 | 240 |  |  |  |  |
| 30 |  |  |  |  |  |  |  |  |  |  | 0.81 |  | 36.5 |
| 31 | 9.7 | 0.01 |  |  |  |  |  | 0.06 | 51.2 |  | 0.05 |  |  |
| 32 | 1.96 |  |  |  |  |  |  | 0.14 | 54.7 |  | 0.44 |  |  |
| 33 | 3.05 |  |  |  |  |  |  |  | 60.2 |  | 0.92 |  |  |
| 34 | 0.57 | 0.07 |  |  |  |  |  | 0.35 | 54.3 |  |  |  |  |
| 35 |  |  |  |  |  |  |  | 9.8 | 37.2 |  | 0.45 |  |  |
| 36 |  | 0.06 |  |  |  |  |  | 5.4 | 83.8 |  | 21.5 |  |  |
| 37 |  |  |  |  |  |  |  | 9.1 | 25.5 |  |  |  | 74.5 |
| 38 |  | 0.07 |  |  |  |  |  | 0.13 | 37.1 |  | 0.81 |  |  |
| 39 |  |  |  |  |  | 98.0 |  | 0.32 | 0.97 |  | 1.31 |  | 0.24 |
| 40 |  |  |  |  |  | 92.8 |  | 1.43 | 0.95 |  | 0.93 |  | 0.93 |
| 41 |  |  |  |  |  |  |  | 0.07 | 1.01 |  | 0.09 |  | 0.13 |
| 42 |  |  |  |  |  |  |  | 0.10 | 1.30 |  | 0.25 |  | 0.12 |
| 43 |  |  |  |  |  |  |  | 0.07 | 1.19 |  | 0.29 |  | 0.08 |
| 44 |  |  |  |  |  |  |  | 0.08 | 1.71 |  | 0.27 |  |  |
| 45 |  |  |  |  |  |  |  | 0.64 | 4.40 |  | 0.16 |  |  |
| 46 |  |  |  |  |  | 96.5 |  | 0.09 | 1.63 |  | 0.82 |  |  |
| 47 |  |  |  |  |  |  |  | 0.13 | 2.33 |  | 0.10 |  |  |
| 48 |  |  |  |  |  |  |  | 0.13 | 2.34 |  | 0.05 |  |  |
| 49 |  |  |  |  |  |  |  |  |  |  | 0.09 |  | 0.11 |
| 50 |  |  |  |  |  | 83.6 |  | 0.20 | 1.27 |  | 0.35 |  | 27.2 |
| 51 |  |  |  |  |  | 111 |  | 0.08 | 1.51 |  | 1.09 |  | 0.08 |
| 52 |  |  |  |  |  |  |  | 0.16 | 1.24 |  | 0.29 |  | 0.11 |
| 53 |  |  |  |  |  | 87.0 |  | 0.24 | 0.95 |  | 1.30 |  | 0.31 |
| 54 |  |  |  |  |  | 99.5 |  | 0.12 | 1.89 |  | 0.75 |  | 2.00 |
| 55 |  |  |  |  |  | 86.1 |  | 2.52 | 6.0 |  | 3.75 |  |  |
| 56 |  |  |  |  |  |  |  |  | 2.79 |  | 6.6 |  | 0.92 |
| 57 |  |  |  |  |  |  |  | 0.96 | 9.7 |  | 1.88 |  |  |
| 58 |  |  |  |  |  |  |  |  | 3.07 |  | 219 |  |  |
| 59 |  |  |  |  |  |  |  |  |  |  | 6.3 |  |  |
| 60 |  |  |  |  |  |  |  |  |  |  | 1.43 |  |  |
| 61 |  |  |  |  | 33.5 |  |  |  |  |  | 1.43 |  |  |
| 62 |  |  |  |  | 33.5 |  |  |  | 1.78 |  | 2.02 |  |  |
| 63 |  |  |  |  |  |  |  | 0.47 | 3.18 |  | 0.51 |  |  |
| 64 |  |  |  |  |  |  |  | 0.24 | 4.11 |  | 0.95 |  |  |
| 65 |  |  |  |  |  |  |  |  | 5.4 |  |  |  |  |
| 66 |  |  |  |  |  |  |  | 0.94 | 5.9 |  | 488 |  |  |
| 67 |  |  |  |  |  |  |  | 4.29 | 2.44 |  | 167 |  | 8.6 |
| 68 |  |  |  |  |  |  |  | 3.42 | 18.4 |  | 77.5 |  | 53.2 |
| 69 |  |  |  |  |  |  |  | 1.39 | 10.9 |  | 98.9 |  |  |

1. **Concentration of pesticides detected in Pakchoi samples bought from Local markets.**

| **Sample No.** | **Pesticide Concentration (ppb)** | | | | | | | | | | | | |
| --- | --- | --- | --- | --- | --- | --- | --- | --- | --- | --- | --- | --- | --- |
|  | **Carba-ryl**  **(50)*** | **Carbo-furan**  **(20)*** | **Chloro-thalonil** | **Chlor-pyrifos**  **(1000)*** | **λ-Cyha-lothrin**  **(1000)*** | **Cyper-methrin**  **(1000)*** | **Delta-methrin** | **Dia-zinon**  **(50)*** | **Dime-thoate**  **(20)*** | **Mala-thion** | **Meta-laxyl**  **(50)*** | **Metho-myl** | **Profe-nofos**  **(50)*** |
| 1 | 5.5 |  |  | 96.6 |  |  |  | 1.30 | 57.4 |  | 8.8 |  | 24.3 |
| 2 |  | 1.05 |  |  |  |  |  | 0.97 | 46.2 |  | 5.9 |  | 6.9 |
| 3 |  |  |  |  | 104 | 92.5 |  |  | 36.2 |  | 3.22 |  |  |
| 4 |  |  |  |  |  |  |  |  | 32.8 |  | 6.7 |  | 0.59 |
| 5 |  | 0.19 |  |  |  |  |  | 2.63 | 74.2 |  | 2.25 |  |  |
| 6 |  |  |  |  |  |  |  | 1.85 | 185 |  | 4.45 |  |  |
| 7 |  |  |  |  |  |  |  | 1.37 | 208 |  | 2.34 |  |  |
| 8 |  |  |  |  |  | 2785 |  | 2.13 | 207 |  | 3.58 |  |  |
| 9 |  |  |  |  |  |  |  | 0.67 | 86.3 |  | 8.1 |  |  |
| 10 | 7.2 |  |  |  |  | 298 |  | 5.8 | 415 |  | 6170 |  |  |
| 11 |  |  |  |  |  | 469 |  | 3.34 | 309 |  | 3.67 |  |  |
| 12 |  |  |  |  |  | 978 |  | 4.57 | 148 |  | 8.8 |  |  |
| 13 |  |  |  |  |  |  |  | 0.59 | 27.6 |  | 5.4 |  |  |
| 14 |  |  |  |  |  | 897 |  | 2.40 | 30.1 |  | 3.81 |  |  |
| 15 |  |  |  |  |  |  |  |  | 9.8 |  | 4.03 |  |  |
| 16 |  |  |  |  |  |  |  |  | 185 |  | 4.45 |  |  |
| 17 |  |  |  |  |  |  |  |  | 2.63 |  | 1.66 |  |  |
| 18 |  |  |  |  |  |  |  |  | 11.9 |  | 0.46 |  |  |
| 19 |  |  |  |  |  |  |  |  | 4.28 |  | 0.86 |  |  |
| 20 |  |  |  | 325 |  | 203 |  | 0.69 | 4.93 |  | 0.58 |  |  |
| 21 |  |  |  |  |  |  |  |  | 10.5 |  | 0.77 |  |  |
| 22 |  |  |  |  |  |  |  |  | 4.20 |  | 0.95 |  | 3.72 |
| 23 |  |  |  |  |  |  |  |  | 40.8 |  | 0.69 |  |  |
| 24 |  |  |  |  |  | 4846 |  |  | 3.48 |  | 1.15 |  |  |
| 25 |  |  |  |  |  |  |  |  | 31.1 |  |  |  |  |
| 26 |  |  |  |  |  |  |  |  |  |  |  |  |  |
| 27 |  |  |  |  |  |  |  |  | 3.66 |  | 0.16 |  |  |
| 28 |  |  |  |  |  |  |  |  | 21.6 |  | 0.23 |  |  |
| 29 |  |  |  |  |  |  |  |  | 5.8 |  | 0.41 |  |  |
| 30 |  |  |  |  |  |  |  |  | 10.6 |  | 0.43 |  |  |
| 31 |  | 0.25 |  |  |  |  |  | 3.75 | 22.3 |  | 26.3 |  |  |
| 32 |  |  |  |  |  |  |  |  | 34.0 |  | 19.2 |  |  |
| 33 |  | 8.66 |  |  | 21.7 |  |  |  | 32.4 |  | 113 |  |  |
| 34 |  | 0.91 |  | 15.5 |  |  |  | 2.59 | 51.1 |  | 68.4 |  |  |
| 35 |  | 1.00 |  |  |  |  |  | 4.01 | 41.2 |  | 84.6 |  | 7016 |
| 36 |  | 5.43 |  |  |  |  |  |  | 17.9 |  | 74.0 |  | 10.4 |
| 37 |  |  |  |  |  |  |  |  | 9.8 |  | 18.5 |  |  |
| 38 |  |  |  |  |  |  |  |  | 38.9 |  | 22.1 |  |  |
| 39 |  |  |  |  |  |  |  | 0.62 | 24.5 |  | 12.2 |  |  |
| 40 |  |  |  |  |  |  |  | 0.47 | 18.7 |  | 8.0 |  |  |
| 41 |  |  |  |  |  |  |  | 2.96 | 14.9 |  | 29.4 |  |  |
| 42 |  |  |  |  |  |  |  | 1.43 | 5.9 |  | 14.9 |  |  |
| 43 |  |  |  |  |  |  |  | 2.42 | 27.2 |  | 17.8 |  | 290 |
| 44 | 514 |  |  | 2339 |  | 784 |  | 0.76 | 5.3 |  | 5.5 |  | 0.53 |
| 45 |  | 0.43 |  |  |  |  |  | 1.81 | 152 |  | 17.8 |  | 1.31 |
| 46 |  |  |  |  |  |  |  | 0.59 | 34.5 |  | 9.6 |  |  |
| 47 |  |  |  |  |  |  |  | 2.94 | 35.7 |  | 19.0 |  |  |
| 48 |  |  |  |  |  |  |  | 0.86 | 333 |  | 23.3 |  |  |
| 49 |  |  |  |  |  |  |  |  | 38.2 |  | 14.1 |  |  |
| 50 |  |  |  |  |  |  |  | 1.47 | 25.6 |  | 77.9 |  |  |
| 51 |  |  |  |  |  |  |  | 1.38 | 13.0 |  | 88.2 |  |  |
| 52 |  |  |  | 4656 |  |  |  | 0.70 | 17.6 |  | 35.9 |  |  |
| 53 |  |  |  |  |  |  |  |  | 32.0 |  | 68.8 |  |  |
| 54 |  |  |  |  |  |  |  | 1.18 | 14.4 |  | 36.4 |  |  |
| 55 |  |  |  |  |  | 156 |  | 1.53 | 16.3 |  | 625 |  |  |
| 56 |  | 0.71 |  |  |  |  |  | 1.42 | 185 |  | 49.5 |  |  |
| 57 |  |  |  |  |  |  |  | 0.28 | 1.65 |  | 61.9 |  |  |
| 58 |  |  |  |  |  |  |  | 0.41 | 1.68 |  | 71.3 |  |  |
| 59 |  |  |  | 24.5 |  | 72.5 |  | 0.26 | 3.16 |  | 121 |  |  |
| 60 |  |  |  | 29.8 |  | 84.7 |  | 0.52 | 5.7 |  | 89.7 |  |  |
| 61 |  |  |  |  |  |  |  |  |  |  | 1.00 |  |  |
| 62 |  |  |  |  |  |  |  |  |  |  | 2.00 |  |  |
| 63 |  |  |  |  |  |  |  |  |  |  | 2.00 |  |  |

**(3) Concentration of pesticides detected in Morning Glory samples bought from Local markets.**

| **Sample No.** | **Pesticide Concentration (ppb)** | | | | | | | | | | | | |
| --- | --- | --- | --- | --- | --- | --- | --- | --- | --- | --- | --- | --- | --- |
|  | **Carba-ryl**  **(10)*** | **Carbo-furan**  **(10)*** | **Chloro-thalonil**  **(10)*** | **Chlor-pyrifos**  **(50)*** | **λ-Cyha-lothrin**  **(20)*** | **Cyper-methrin**  **(700)*** | **Delta-methrin** | **Dia-zinon**  **(10)*** | **Dime-thoate**  **(20)*** | **Mala-thion**  **(20)*** | **Meta-laxyl**  **(2000)*** | **Metho-myl**  **(20)*** | **Profe-nofos**  **(10)*** |
| 1 |  |  |  |  |  |  |  | 1.21 | 27.3 |  | 5.45 |  | 16.0 |
| 2 |  |  |  |  |  |  |  | 10.6 | 26.2 |  | 8.2 |  | 9.1 |
| 3 |  | 1.05 |  |  |  |  |  |  | 27.1 |  | 2.42 |  |  |
| 4 |  |  |  |  |  |  |  |  | 26.9 |  | 1.26 |  |  |
| 5 |  |  |  |  |  |  |  | 1.16 | 26.0 |  | 0.64 |  |  |
| 6 |  |  |  |  |  |  |  | 4.44 | 37.6 |  | 6.5 |  |  |
| 7 |  |  |  |  |  |  |  | 0.61 | 41.6 |  | 2.25 |  |  |
| 8 |  | 5.2 |  |  |  |  |  | 1.48 | 30.4 |  | 1.91 |  |  |
| 9 |  |  |  |  |  |  |  | 2.38 | 35.2 |  | 4.43 |  |  |
| 10 |  |  |  |  |  |  |  | 2.43 | 36.2 |  | 4.21 |  |  |
| 11 |  | 3.72 |  |  |  |  |  | 0.68 | 29.0 |  | 5.6 |  |  |
| 12 |  |  |  |  |  |  |  | 0.62 | 5.22 |  | 5.32 |  |  |
| 13 |  | 2.48 |  |  |  |  |  | 0.62 | 0.04 |  | 4.50 |  |  |
| 14 |  |  |  |  |  |  |  |  |  |  | 2.05 |  |  |
| 15 |  |  |  |  |  |  |  |  |  |  | 1.61 |  |  |
| 16 |  |  |  |  |  | 2099 |  |  | 2.55 |  | 1.94 |  |  |
| 17 |  |  |  |  |  |  |  |  |  |  | 1.00 |  |  |
| 18 |  |  |  |  |  |  |  |  |  |  | 0.54 |  |  |
| 19 |  |  |  |  |  |  |  |  | 2.12 |  | 0.83 |  |  |
| 20 |  |  |  |  |  | 89.7 |  |  | 3.60 |  | 0.45 | 0.06 |  |
| 21 |  |  |  |  |  |  |  |  |  |  |  |  |  |
| 22 |  |  |  |  |  |  |  |  | 9.52 |  | 0.49 |  |  |
| 23 |  |  |  |  |  | 1304 |  |  |  |  | 0.20 |  |  |
| 24 |  |  |  |  |  |  |  |  | 1.53 |  | 0.12 |  |  |
| 25 |  |  |  |  |  | 4031 |  |  | 4.08 |  | 0.31 |  |  |
| 26 |  |  |  |  |  |  |  |  | 3.74 |  | 0.28 |  |  |
| 27 |  |  |  |  |  |  |  |  | 2.64 |  |  |  |  |
| 28 |  |  |  |  |  |  |  |  | 3.17 |  |  |  |  |
| 29 |  | 5.1 |  |  |  |  |  |  | 3.34 |  | 11.1 | 6.6 |  |
| 30 |  | 4.34 |  |  |  |  |  |  | 0.72 |  | 5.6 | 5.7 |  |
| 31 |  |  |  |  |  |  |  |  | 0.71 |  | 15.3 |  |  |
| 32 |  |  |  |  |  |  |  |  | 19.8 |  | 28.5 |  |  |
| 33 |  |  |  |  |  |  |  |  | 13.1 |  | 26.3 |  |  |
| 34 |  |  |  |  |  |  |  |  | 0.46 |  | 8.5 |  |  |
| 35 |  |  |  |  |  |  |  |  | 3.11 |  | 10.2 |  |  |
| 36 |  |  |  |  |  |  |  |  | 1.29 |  | 5.7 |  |  |
| 37 |  | 0.07 |  |  |  |  |  |  | 6.1 |  | 297 |  |  |
| 38 |  | 0.90 |  |  |  |  |  |  | 32.3 |  | 55.2 |  |  |
| 39 |  | 0.35 |  |  |  |  |  |  | 6.1 |  | 97.5 |  |  |
| 40 |  | 1.68 |  |  |  | 352 |  |  | 7.6 |  | 92.0 |  |  |
| 41 |  |  |  |  |  |  |  |  | 32.7 |  | 83.2 |  |  |
| 42 |  | 0.56 |  |  |  |  |  | 6.1 | 249 |  | 82.2 |  |  |
| 43 |  | 0.64 |  |  |  | 709 |  | 5.6 | 71.8 |  | 73.8 |  |  |
| 44 |  |  |  |  |  |  |  | 1.06 | 43.6 |  | 66.7 |  |  |
| 45 |  | 0.39 |  |  |  |  |  | 1.42 | 7.7 |  | 68.0 |  |  |
| 46 |  |  |  |  |  |  |  | 0.46 | 20.0 |  | 60.2 |  |  |
| 47 |  | 1.03 |  |  |  |  |  | 0.97 | 17.9 |  | 92.8 |  |  |
| 48 |  | 2.61 |  |  |  |  |  | 1.28 | 166 |  | 275 |  |  |
| 49 |  | 6.7 |  |  |  |  |  | 1.16 | 85.6 |  | 275 |  |  |
| 50 |  | 4.31 |  | 34.4 |  |  |  |  | 9.7 |  | 179 |  |  |
| 51 |  |  |  |  |  |  |  | 0.61 | 108 |  | 235 |  |  |
| 52 |  | 1.00 |  |  |  |  |  | 0.74 | 10.4 |  | 192 |  |  |
| 53 |  | 1.32 |  |  |  |  |  | 0.78 | 19.9 |  | 130 |  |  |
| 54 |  | 0.94 |  |  |  |  |  | 1.58 | 10.3 |  | 178 |  |  |
| 55 |  |  |  |  |  |  |  | 2.29 | 132 |  | 279 |  |  |
| 56 |  |  |  |  |  |  |  | 0.38 | 174 |  | 39.3 |  |  |
| 57 |  | 1.94 |  |  |  |  |  |  | 31.2 |  | 208 |  |  |
| 58 |  |  |  |  |  |  |  | 0.71 | 13.7 |  | 121 |  |  |
| 59 |  |  |  |  |  |  |  |  | 93.6 |  | 61.5 |  |  |
| 60 |  |  |  |  |  |  |  |  | 231 |  | 139 |  |  |
| 61 |  |  |  |  |  |  |  |  | 86.7 |  | 105.6 |  |  |
| 62 |  |  |  |  |  |  |  | 0.5 | 38.8 |  | 88.3 |  |  |
| 63 |  |  |  |  |  |  |  | 0.3 | 2.8 |  | 74.2 |  |  |
| 64 |  |  |  |  |  |  |  | 0.4 | 4.1 |  | 14.7 |  |  |
| 65 |  |  |  |  |  |  |  | 0.6 | 4.6 |  | 12.7 |  |  |
| 66 |  | 0.15 |  | 111320 | 1402 | 16998 |  | 1.0 | 84.6 | 148 | 5.4 | 11184 | 1504 |
| 67 |  |  |  | 5.0 |  | 55.05 |  | 1.40 | 1.37 |  | 2.78 |  |  |
| 68 |  |  |  | 5.6 |  |  |  | 2.07 | 3.74 |  | 2.42 |  |  |
| 69 |  |  |  | 5.9 |  |  |  | 1.56 | 8.3 |  | 5.3 |  |  |
| 70 |  |  |  |  |  |  |  | 0.54 | 1.53 |  | 2.09 |  |  |
| 71 |  |  |  |  |  |  |  | 0.93 | 1.55 |  | 3.88 |  |  |
| 72 |  | 2.89 |  |  |  |  |  | 1.01 | 1.92 |  | 4.99 |  |  |
| 73 |  | 4.02 |  |  |  |  |  | 1.29 | 4.10 |  | 3.32 |  |  |
| 74 |  |  |  |  |  |  |  | 1.68 | 4.66 |  | 5.0 |  |  |
