# Supplementary material for: Food safety in Thailand 4: comparison of pesticide residues found in three commonly consumed vegetables purchased from local markets and supermarkets in Thailand
Source: PeerJ. 2016 Sep 1;4:e2432. doi: 10.7717/peerj.2432 (PMC5012412; doi:10.7717/peerj.2432)
Supplement: Data S2 [file peerj-04-2432-s002.docx]

**Supplementary Data II**

**(1) Concentration of pesticides detected in Chinese kale samples bought from Supermarkets.**

| **Sample No.** | **Pesticide Concentration (ppb)** | | | | | | | | | | | | |
| --- | --- | --- | --- | --- | --- | --- | --- | --- | --- | --- | --- | --- | --- |
|  | **Carba-ryl**  **(50)*** | **Carbo-furan (20)*** | **Chloro-thalonil (4000)*** | **Chlor-pyrifos (50)*** | **λ-Cyha-lothrin (1000)*** | **Cyper-methrin (1000)*** | **Delta-methrin (500)*** | **Dia-zinon (50)*** | **Dime-thoate (20)*** | **Mala-thion (3000)*** | **Meta-laxyl (2000)*** | **Metho-myl** | **Profe-nofos (10)*** |
| 1 |  |  |  |  |  |  |  | 0.02 |  |  | 0.12 |  |  |
| 2 |  |  |  |  |  |  |  |  |  |  |  |  |  |
| 3 |  |  |  |  |  |  |  |  |  |  | 0.02 |  |  |
| 4 |  |  |  |  |  |  |  |  | 5.5 |  |  |  |  |
| 5 |  |  |  |  |  |  |  |  | 41.4 |  |  |  |  |
| 6 |  |  | 136 |  |  |  |  | 10.5 | 15.7 |  | 0.19 |  | 259 |
| 7 |  |  | 36.4 |  |  |  |  | 0.57 | 20.9 |  | 0.65 |  |  |
| 8 | 11.1 | 0.53 |  |  |  | 183 |  |  | 9.1 |  | 1.51 |  |  |
| 9 | 5.3 |  |  |  |  |  |  |  | 31.4 |  | 0.48 |  |  |
| 10 | 0.26 | 0.01 |  |  |  |  |  |  | 1.91 |  |  |  | 60.8 |
| 11 |  |  |  |  |  | 83.3 |  | 0.10 | 0.98 |  | 0.21 |  | 0.47 |
| 12 |  |  |  |  |  | 83.1 |  | 0.14 |  |  | 0.07 |  | 0.13 |
| 13 |  |  |  |  |  |  |  | 0.51 | 2.34 |  | 14.5 |  | 31.9 |
| 14 |  |  |  |  |  | 48.1 |  | 1.41 | 37.8 |  | 24.3 |  | 22.2 |
| 15 |  |  |  |  |  |  |  | 3.06 | 50.8 |  | 11.0 |  | 25.5 |
| 16 |  |  |  |  |  |  |  | 1.24 | 8.2 |  | 3.23 |  | 27.0 |
| 17 |  |  |  |  |  |  |  | 2.10 | 43.9 |  | 13.4 |  | 41.1 |
| 18 |  |  |  |  |  |  |  | 6.6 | 64.7 |  | 17.7 |  | 31.1 |
| 19 |  |  |  |  |  |  |  |  | 2.85 |  | 7.2 |  | 1.14 |
| 20 |  |  |  |  |  |  |  |  | 2.12 |  | 7.3 |  |  |
| 21 |  |  |  |  |  |  |  |  | 6.3 |  | 6.3 |  | 1.40 |
| 22 |  |  |  |  |  |  |  | 1.19 | 10.8 |  | 1.49 |  | 34.8 |
| 23 |  |  |  | 1019 |  |  |  | 0.94 | 7.8 |  | 1.67 |  | 1.05 |
| 24 |  |  |  |  |  |  |  | 0.74 | 6.6 |  | 22.9 |  | 9.78 |
| 25 |  |  |  |  |  | 88.3 |  | 0.89 | 8.7 |  | 1.66 |  |  |
| 26 |  |  |  |  |  | 23.5 |  | 8.2 | 34.5 |  | 7.59 |  |  |
| 27 |  |  |  |  |  |  |  | 3.18 | 18.5 |  | 3.64 |  |  |
| 28 |  |  |  |  |  |  |  | 4.57 | 32.8 |  | 9.5 |  |  |
| 29 | 606 |  |  |  |  |  | 152 | 3.17 | 12.6 |  | 9.0 |  | 2095 |
| 30 |  | 763 |  |  | 8.4 | 183514 |  | 1.89 | 29.2 |  | 8.5 |  | 2.20 |
| 31 |  |  |  |  |  |  |  | 1.26 | 14.2 |  | 15.4 |  |  |
| 32 |  |  |  |  |  |  |  |  | 1.71 |  | 1.77 |  |  |
| 33 |  |  |  |  |  | 1556 |  | 1.42 | 9.8 |  | 11.7 |  |  |
| 34 |  |  |  |  |  |  |  |  | 12.9 |  | 4.60 |  |  |
| 35 |  |  |  |  |  |  |  |  |  |  | 3.25 |  |  |
| 36 |  |  |  |  |  |  |  | 0.60 | 2.95 |  | 13.6 |  | 988 |
| 37 |  |  |  |  |  | 8736 |  | 2.94 | 1.77 |  | 0.52 |  |  |
| 38 |  |  |  |  |  |  |  | 0.36 | 1.68 |  | 0.81 |  |  |
| 39 |  |  |  |  |  |  |  |  | 5.2 |  | 0.34 |  |  |
| 40 |  |  |  |  |  |  |  |  | 1.92 |  | 0.51 |  |  |
| 41 |  |  |  |  |  |  |  | 1.98 | 7.0 |  | 39.2 |  |  |
| 42 |  |  |  |  |  |  |  |  | 2.90 |  | 0.21 |  |  |
| 43 |  |  |  |  |  |  |  | 0.23 | 3.21 |  | 0.15 |  |  |
| 44 |  |  |  |  |  |  |  |  | 1.32 |  | 15.8 |  | 139 |
| 45 | 88.1 |  |  |  |  |  |  | 7.8 | 1.95 |  | 52.8 |  |  |
| 46 |  |  |  |  |  |  |  | 1.17 | 1.08 |  | 13.4 |  |  |
| 47 |  | 0.96 |  |  |  |  |  | 3.78 | 1.15 |  | 10.7 |  | 3.50 |
| 48 |  |  |  |  |  |  |  | 3.92 | 6.4 |  | 109 |  |  |
| 49 |  |  |  |  |  |  |  | 4.95 | 15.4 |  | 68.2 |  |  |
| 50 |  | 0.87 |  |  |  |  |  | 6.7 | 17.7 |  | 181 |  |  |
| 51 |  | 0.32 |  |  |  |  |  | 5.6 | 16.3 |  | 141 |  |  |
| 52 |  | 0.24 |  |  |  |  |  | 3.07 | 11.4 |  | 49.7 |  |  |
| 53 |  | 0.18 |  |  |  |  |  | 1.99 | 4.55 |  | 93.8 |  |  |
| 54 |  |  |  |  |  |  |  | 0.45 | 4.67 |  | 25.0 |  |  |
| 55 |  |  |  |  |  | 25.5 |  | 0.91 | 6.1 |  | 44.9 |  |  |
| 56 |  |  |  |  |  |  |  | 2.77 | 0.93 |  | 49.5 |  |  |
| 57 |  |  |  |  |  |  |  | 0.50 | 0.77 |  | 27.6 |  |  |
| 58 |  |  |  |  |  |  |  | 1.83 | 15.8 |  | 14.8 |  |  |
| 59 |  |  |  |  |  |  |  | 2.23 | 2.57 |  | 22.4 |  |  |
| 60 |  |  |  |  |  |  |  | 6.0 | 15.6 |  | 10.1 |  |  |
| 61 |  |  |  |  |  |  |  | 0.66 | 1.20 |  | 11.8 |  |  |
| 62 |  |  |  |  |  |  |  |  |  |  | 0.65 |  |  |
| 63 | 17.3 |  |  | 6.7 |  |  |  | 1.0 | 6.7 |  | 2.76 |  |  |
| 64 |  |  |  | 3.77 |  |  |  | 0.58 | 23.2 |  | 4.49 |  |  |
| 65 | 2.74 |  |  | 5.1 |  |  |  | 1.08 |  |  | 2.75 |  |  |
| 66 | 6.5 |  |  | 8.1 |  |  |  | 1.37 | 6.0 |  | 3.25 |  |  |
| 67 | 6.8 |  |  | 5.2 |  |  |  | 1.62 | 6.2 |  | 3.69 |  |  |
| 68 | 8.7 |  |  | 3.75 |  |  |  | 1.06 |  |  | 4.5 |  |  |

**(2) Concentration of pesticides detected in Pakchoi samples bought from Supermarkets.**

| **Sample No.** | **Pesticide Concentration (ppb)** | | | | | | | | | | | | |
| --- | --- | --- | --- | --- | --- | --- | --- | --- | --- | --- | --- | --- | --- |
|  | **Carba-ryl**  **(50)*** | **Carbo-furan**  **(20)*** | **Chloro-thalonil** | **Chlor-pyrifos**  **(1000)*** | **λ-Cyha-lothrin**  **(1000)*** | **Cyper-methrin**  **(1000)*** | **Delta-methrin** | **Dia-zinon**  **(50)*** | **Dime-thoate**  **(20)*** | **Mala-thion** | **Meta-laxyl**  **(50)*** | **Metho-myl** | **Profe-nofos**  **(50)*** |
| 1 |  |  |  |  |  |  |  | 1.42 | 145 |  | 7.2 |  | 16.9 |
| 2 |  |  |  |  |  |  |  | 1.21 | 137 |  | 10.8 |  | 30.8 |
| 3 |  |  |  |  |  |  |  | 1.94 | 25.6 |  | 36.4 |  | 13.4 |
| 4 |  |  |  |  |  |  |  | 1.99 | 41.4 |  | 6.0 |  | 7.7 |
| 5 |  |  |  |  |  | 120 |  |  | 38.4 |  | 6.9 |  |  |
| 6 |  |  |  |  |  |  |  |  | 40.0 |  | 1.84 |  | 0.52 |
| 7 |  |  |  |  |  |  |  |  | 26.5 |  | 4.46 |  |  |
| 8 |  |  |  |  |  | 174 |  | 0.70 | 44.5 |  | 1.14 |  |  |
| 9 |  |  |  |  |  |  |  | 0.74 | 36.7 |  | 1.31 |  |  |
| 10 |  | 0.12 |  |  |  |  |  | 7.2 | 45.9 |  | 1.90 |  | 36.1 |
| 11 |  |  |  |  |  |  |  | 0.59 | 35.8 |  | 1.08 |  |  |
| 12 |  |  |  |  |  |  |  | 2.23 | 93.2 |  | 4.65 |  |  |
| 13 |  |  |  | 4335 |  | 2654 |  |  | 56.8 |  | 4.81 |  |  |
| 14 |  |  |  |  |  |  |  |  | 11 |  | 4.26 |  |  |
| 15 |  |  |  |  |  |  |  | 2.44 | 29.7 |  | 5.3 |  |  |
| 16 |  |  |  |  |  |  |  |  | 6.1 |  | 4.29 |  |  |
| 17 |  |  |  |  |  |  |  | 2.59 | 112 |  | 13.9 |  |  |
| 18 |  |  |  |  |  |  |  | 5.3 | 33.2 |  | 8.1 |  |  |
| 19 |  |  |  |  |  | 71.0 |  |  | 14.3 |  | 1.90 |  |  |
| 20 |  |  |  |  |  |  |  |  | 1.42 |  | 2.48 |  |  |
| 21 |  |  |  |  |  |  |  | 3.55 | 58.2 |  | 7.9 |  |  |
| 22 |  |  |  |  |  | 38.9 |  |  | 12.6 |  | 2.73 |  |  |
| 23 |  |  |  |  |  |  |  |  | 3.38 |  | 4.63 |  |  |
| 24 | 1.03 |  |  |  |  |  |  |  |  |  | 0.42 |  |  |
| 25 |  |  |  |  |  |  |  |  | 6.25 |  | 1.95 |  |  |
| 26 |  |  |  |  |  |  |  |  | 3.23 |  | 0.78 |  |  |
| 27 |  |  |  |  |  |  |  |  | 1.73 |  | 0.35 |  |  |
| 28 |  |  |  |  |  | 19.2 |  |  | 4.75 |  | 0.36 |  |  |
| 29 |  |  |  |  |  |  |  |  | 8.2 |  | 0.60 |  |  |
| 30 |  |  |  |  |  |  |  |  | 11.8 |  | 0.40 |  |  |
| 31 |  |  |  |  |  |  |  |  | 1.37 |  |  |  |  |
| 32 |  |  |  |  |  | 71292 |  | 4.31 | 19.6 |  | 26.1 |  | 80.9 |
| 33 | 17.1 | 3.46 |  |  |  | 7304.0 |  | 5.8 | 15.5 |  | 34.1 |  |  |
| 34 | 34.8 | 1.84 |  |  |  | 2971 |  | 7.8 | 35.7 |  | 28.7 |  |  |
| 35 |  |  |  |  |  | 29726 |  | 4.01 | 39.0 |  | 1060 |  |  |
| 36 |  |  |  |  |  |  |  | 1.92 | 43.1 |  | 190 |  |  |
| 37 |  | 7.0 |  |  |  |  |  | 3.58 | 40.0 |  | 112.2 |  |  |
| 38 |  | 1.62 |  |  |  |  |  |  | 18.6 |  | 98.3 |  |  |
| 39 |  | 3.26 |  |  |  |  |  | 3.24 | 16.8 |  | 73.1 |  |  |
| 40 |  |  |  |  |  |  |  | 6.5 | 14.06 |  | 235.7 |  |  |
| 41 |  | 8.1 |  |  |  |  |  | 9.0 | 8.3 |  | 118 |  |  |
| 42 |  | 0.46 |  |  |  |  |  | 1.15 | 4.45 |  | 68.3 |  |  |
| 43 |  | 1.57 |  |  |  | 148 |  | 2.88 | 19.6 |  | 144 |  |  |
| 44 |  | 0.93 |  |  |  | 258 |  |  | 7.8 |  | 64.0 |  |  |
| 45 |  |  |  |  |  |  |  | 1.39 | 3.45 |  | 46.8 |  |  |
| 46 |  | 1.31 |  |  |  |  |  | 1.65 | 16.7 |  | 25.2 |  |  |
| 47 |  | 0.75 |  |  |  |  |  | 1.69 | 40.2 |  | 14.2 |  | 976 |
| 48 |  | 1.17 |  |  |  |  |  | 1.49 | 14.7 |  | 8.3 |  |  |
| 49 |  |  |  |  |  |  |  | 1.99 | 59.5 |  | 24.8 |  |  |
| 50 |  | 4.79 |  |  |  |  |  | 4.30 | 12.6 |  | 57.1 |  |  |
| 51 |  |  |  |  |  |  |  | 1.75 | 1.50 |  | 22.5 |  |  |
| 52 |  |  |  |  |  |  |  | 0.86 | 1.45 |  | 36.6 |  |  |
| 53 |  |  |  |  |  |  |  |  |  |  | 18.3 |  |  |
| 54 | 45.6 |  |  | 1.87 |  |  |  |  |  |  | 1.12 |  |  |
| 55 |  |  |  | 2.18 |  |  |  | 0.84 | 2.80 |  | 2.52 |  |  |
| 56 | 8.9 | 3.45 |  | 2.78 |  |  |  | 0.26 | 1.54 |  | 2.53 |  |  |
| 57 |  |  |  | 2.26 |  | 17.9 |  | 1.02 | 2.06 |  | 2.56 |  |  |
| 58 |  |  |  | 2.41 |  |  |  | 1.05 |  |  | 6.5 |  |  |
| 59 |  | 2.28 |  | 2.18 |  |  |  | 0.63 |  |  | 6.4 |  |  |
| 60 | 19.1 | 1.27 |  | 3.02 |  |  |  | 0.66 |  |  | 5.8 |  |  |
| 61 | 29.2 |  |  | 3.51 |  |  |  | 1.19 |  |  | 3.31 |  |  |
| 62 | 17.2 | 0.99 |  | 2.53 |  |  |  | 1.22 |  |  | 4.94 |  |  |

**(3) Concentration of pesticides detected in Morning Glory samples bought from Supermarkets.**

| **Sample No.** | **Pesticide Concentration (ppb)** | | | | | | | | | | | | |
| --- | --- | --- | --- | --- | --- | --- | --- | --- | --- | --- | --- | --- | --- |
|  | **Carba-ryl**  **(10)*** | **Carbo-furan**  **(10)*** | **Chloro-thalonil**  **(10)*** | **Chlor-pyrifos**  **(50)*** | **λ-Cyha-lothrin**  **(20)*** | **Cyper-methrin**  **(700)*** | **Delta-methrin** | **Dia-zinon**  **(10)*** | **Dime-thoate**  **(20)*** | **Mala-thion**  **(20)*** | **Meta-laxyl**  **(2000)*** | **Metho-myl**  **(20)*** | **Profe-nofos**  **(10)*** |
| 1 |  | 1.55 |  |  |  |  |  | 2.84 | 32.0 |  | 6.8 |  | 2.96 |
| 2 |  | 1.03 |  |  |  |  |  | 0.62 |  |  | 4.93 |  |  |
| 3 |  | 1.07 |  |  |  |  |  | 4.04 | 25.9 |  | 17.3 |  | 18.5 |
| 4 |  | 0.98 |  |  |  |  |  | 1.77 | 29.8 |  | 17.1 |  |  |
| 5 |  |  |  |  |  |  |  | 3.30 | 39.9 |  | 10.3 |  | 19.0 |
| 6 |  | 1.51 |  |  |  | 329 |  | 0.54 | 25.7 |  | 3.51 |  | 0.20 |
| 7 |  | 0.57 |  |  |  |  |  | 0.37 | 30.3 |  | 3.03 |  |  |
| 8 |  |  |  |  |  |  |  | 0.52 | 25.0 |  | 3.56 |  |  |
| 9 |  | 0.44 |  |  |  |  |  | 0.61 | 25.3 |  | 7.8 |  |  |
| 10 |  |  |  |  |  |  |  | 0.71 | 35.6 |  | 2.67 |  |  |
| 11 |  |  |  |  |  | 329 |  | 1.25 | 31.2 |  | 2.39 |  |  |
| 12 |  |  |  |  |  |  |  | 0.50 | 28.8 |  | 0.54 |  |  |
| 13 |  |  |  |  |  |  |  | 2.68 | 27.0 |  | 1.08 |  |  |
| 14 |  | 1.39 |  |  |  |  |  | 1.19 | 25.2 |  | 0.82 |  |  |
| 15 |  |  |  |  |  | 301 |  | 2.00 | 26.3 |  | 13.8 |  |  |
| 16 |  |  |  |  |  |  |  | 2.20 | 29.9 |  | 11.8 |  |  |
| 17 |  | 8.3 |  |  |  | 4.40 |  | 7.1 | 145 |  | 6.78 |  | 0.80 |
| 18 |  | 5.9 |  |  |  | 56.1 |  | 2.73 | 47.3 |  | 6.4 |  |  |
| 19 |  |  |  |  |  |  |  | 4.77 | 31.6 |  | 29.2 |  |  |
| 20 |  |  |  |  |  | 785 |  | 1.50 | 4.17 |  | 3.29 |  |  |
| 21 |  |  |  |  |  |  |  | 1.62 | 2.95 |  | 1.85 |  |  |
| 22 |  |  |  |  |  | 103.6 |  | 9.22 | 22.3 |  | 12.0 |  |  |
| 23 |  |  |  |  |  |  |  |  |  |  | 3.44 |  |  |
| 24 |  |  |  |  |  |  |  |  |  |  | 3.13 |  |  |
| 25 |  |  |  |  |  |  |  |  | 4.61 |  | 1.93 |  |  |
| 26 |  |  |  |  |  |  |  | 1.38 | 2.41 |  | 2.29 |  |  |
| 27 |  |  |  |  |  |  |  | 1.19 | 1.10 |  | 4.35 |  |  |
| 28 |  |  |  |  |  |  |  |  |  |  | 0.77 |  |  |
| 29 |  |  |  |  |  |  |  |  |  |  |  |  |  |
| 30 |  |  |  |  |  | 14.6 |  |  | 1.98 |  |  |  |  |
| 31 |  |  |  |  |  |  |  | 0.42 | 12.4 |  |  |  |  |
| 32 |  |  |  |  |  |  |  |  |  |  |  |  |  |
| 33 |  |  |  |  |  |  |  |  | 3.47 |  | 16.2 |  |  |
| 34 |  |  |  |  |  | 3425 |  |  | 12.1 |  | 10.3 |  |  |
| 35 |  |  |  |  |  |  |  |  |  |  | 12.0 |  |  |
| 36 |  |  |  | 104 |  | 106 |  | 3.51 | 3.42 |  | 26.3 |  |  |
| 37 |  | 5.5 |  |  |  |  |  | 5.8 | 5.1 |  | 85.4 |  |  |
| 38 |  |  |  |  |  |  |  |  | 10.2 |  | 34.8 |  |  |
| 39 |  |  |  |  |  |  |  |  |  |  | 32.0 |  |  |
| 40 |  | 1.42 |  |  |  |  |  | 1.62 | 20.5 |  | 61.1 |  |  |
| 41 |  |  |  |  |  |  |  | 2.36 | 1.14 |  | 39.8 |  |  |
| 42 |  | 0.35 |  |  |  |  |  | 1.70 | 18.3 |  | 115 |  |  |
| 43 |  | 0.36 |  | 20.7 | 2.79 |  |  | 2.69 | 12.1 |  | 257 |  |  |
| 44 |  | 3.93 |  |  | 1.15 | 1680 |  | 0.94 | 9.7 |  | 38.2 |  |  |
| 45 |  | 2.21 |  |  |  |  |  | 2.93 | 9.2 |  | 89 |  |  |
| 46 |  |  |  |  |  |  |  | 3.00 |  |  | 26.4 |  |  |
| 47 |  |  |  |  |  |  |  | 5.0 | 78.7 |  | 131 |  |  |
| 48 |  |  |  |  |  |  |  |  | 31.9 |  | 45.9 |  |  |
| 49 |  |  |  |  |  | 363 |  | 1.07 | 2.69 |  | 64.1 |  |  |
| 50 |  | 0.13 |  |  |  |  |  | 0.88 | 2.64 |  | 54.1 |  |  |
| 51 |  |  |  | 1.61 |  | 22.8 |  | 0.83 | 4.01 |  | 53.8 |  |  |
| 52 |  |  |  | 4.73 |  |  |  | 1.96 | 4.41 |  | 3.30 |  |  |
| 53 |  |  | 3152 | 3.24 |  |  |  | 1.40 | 1.10 |  | 3.11 |  |  |
| 54 |  |  |  | 3.18 |  |  |  | 1.90 | 1.29 |  | 2.05 |  |  |
| 55 | 12687 |  |  | 3.71 |  | 170 |  | 1.86 | 7.08 |  | 44.8 |  |  |
| 56 |  |  |  | 3.78 |  |  |  | 1.79 |  |  | 10.2 |  |  |
| 57 |  |  |  | 6.4 |  | 722 |  | 0.71 | 6.2 |  | 3.83 |  |  |
| 58 |  |  |  | 6.5 |  | 0.44 |  | 1.65 | 13.3 |  | 5.05 |  |  |
| 59 |  |  |  | 6.5 |  |  |  | 1.69 | 11.3 |  | 4.64 |  |  |
| 60 |  |  |  | 6.6 |  |  |  | 1.91 | 2.30 |  | 2.89 |  |  |
| 61 |  |  |  | 6.0 |  |  |  | 1.96 | 5.3 |  | 3.76 |  |  |
